# Effects of raw and thermally processed spent coffee grounds on *Miscanthus × giganteus* plantation: Data description

**DOI:** 10.1016/j.dib.2025.112432

**Published:** 2026-01-07

**Authors:** Nicole Nawrot, Jacek Kluska

**Affiliations:** a Gdansk University of Technology, Faculty of Civil and Environmental Engineering, Narutowicza 11/12, 80-233, Gdansk, Poland; b Institute of Fluid Flow Machinery, Polish Academy of Sciences, Gdansk, Poland

**Keywords:** *Miscanthus giganteus*, Spent coffee grounds, Biochar, Nutrients, Growth response, Photosynthesis rates

## Abstract

Cultivating *Miscanthus × giganteus* (*M×g*) energy crop on marginal soil supports phytoattenuation and provides high-energy biomass for biofuel production. Improving nutrient-poor soil with low-cost recovered organic amendments, such as spent coffee grounds (SCG) and SCG-derived biochar (BC) offers sustainable benefits. This data article presents the findings from a medium-term greenhouse experiment at the Gdansk University of Technology assessing *M×g* cultivation on marginal soil with SCG and BC amendments into soil. In a pot-scale experiment the medium term-effect on *M×g* biomass growth, photosynthesis parameters, root tissues development, as well as final elemental composition was examined. Soil pH and elemental composition were also determined. As global coffee consumption increases, large quantities of SCG are generated and often landfilled. Their beneficial reuse aligns with circular economy principles and Sustainable Development Goals (SDGs 7 and 13), providing both a short-term nutrient source and a means of improving soil quality and resilience. The article compiles five datasets detailing: (1) *M×g* growth parameters, tissue development, and photosynthetic indices, (2) nutrient and caffeine leaching behaviour; and (3) elemental composition of plants and soils following exposure. These datasets, available in the Bridge of Knowledge Gdansk University of Technology repository, provide a resource for environmental researchers, soil and plant scientists, biochar specialists, and decisionmakers working to restore marginal soil usability. This study promotes sustainable land management by demonstrating how organic wastes and biochar can be combined to improve crop performance, sequester carbon, and reduce nutrient losses while minimizing external fertilizer inputs.

## Graphical abstract

**Figure:**
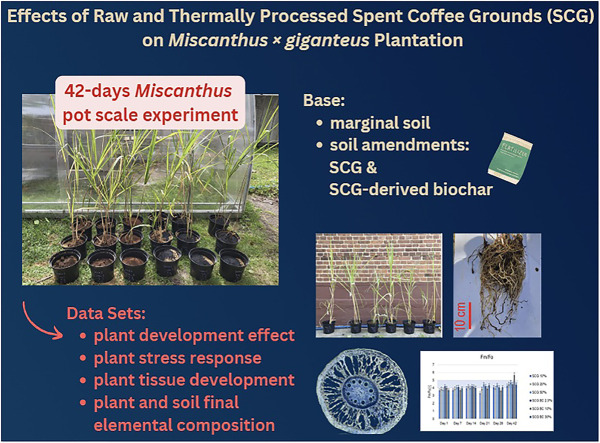

Specifications Table

**Table:** 

Subject	Earth & Environmental Sciences
Specific subject area	Phytoremediation, Plant Science, Soil Science.
Type of data	Table, Image, Raw, Analyzed
Data collection	Data were collected from a greenhouse pot scale experiment with Miscanthus *×* giganteus growing on marginal soil supplemented with dry spent coffee grounds (SCG) and biochar SCG amendments. Spectrophotometer, LC, ICP-OES, XRF, Chlorophyll Content Meter, and fluorimeter set were used.
Data source location	Gdańsk University of Technology campus, Gdańsk, Poland, 54°22′08.9″N 18°36′48.4″E.
Data accessibility	Repository name: Bridge of Knowledge, Gdansk University of Technology Data identification number: Dataset 1: 10.34808/h744–9m76; Dataset 2: 10.34808/h226-gv11; Dataset 3: 10.34808/0yfx-ep80; Dataset 4: 10.34808/r6ax-5b22; Dataset 5: 10.34808/w3p6–0622 Direct URL to data: https://doi.org/10.34808/h744–9m76; https://doi.org/10.34808/0yfx-ep80; https://doi.org/10.34808/h226-gv11; https://doi.org/10.34808/r6ax-5b22; https://doi.org/10.34808/w3p6–0622
Related research article	none

## 1 Value of the Data

• The dataset provides valuable insights into the application potential of organic wastes – specifically spent coffee grounds (SCG) and SCG-derived biochar – in energy biomass cultivation and phytoattenuation processes under temperate climate conditions;
• Researchers can use the data to assess the effects of SCG and SCG-derived biochar (SCG BC) on growth performance, tissue development, and photosynthetic parameters of *Miscanthus × giganteus*;
• The dataset includes quantitative measurements of nutrient and caffeine leaching, offering a reliable basis environmental risk assessment of SCG-based amendments;
• The data support circular economy approaches by demonstrating pathways for the beneficial reuse of large quantities of SCG generated worldwide;
• Environmental management practitioners may use the data to plan strategies for restoring or improving marginal and degraded soils;
• This data article can guide future field-scale applications of waste-derived amendments aimed at improving energy crop productivity while reducing dependency on external fertilizers and minimizing nutrient losses.

## 2 Background

*Miscanthus × giganteus* is a perennial C4 species with rapid growth and efficient photosynthesis, characterized by a high ability to absorb large amounts of CO_2_ from the atmosphere and store carbon in extensive root and rhizome systems. This characteristic is essential for carbon-negative bioenergy systems that support climate-change mitigation strategies [1]. C4 species (e.g. *Miscanthus × giganteus, Saccharum officinarum, Zea mays*) have higher water-use efficiency, reduced photorespiration, and greater nitrogen-use efficiency due to their CO₂-concentrating mechanism. As a result, they use water and nitrogen more efficiently, produce more biomass, and are more adaptable to dry or marginal conditions than C3 species [2].

*Miscanthus* sp. has modest input requirements, making it appropriate for planting on marginal lands (e.g., sandy, nutrient-poor soils, saline soils or other areas with inherent limitations) as well as on abandoned lands (i.e., previously cultivated areas that have been left unused due to socioeconomic or environmental factors). Globally, marginal lands cover 4–6 billion hectares (depending on the criteria used: arid, degraded, or low-fertility areas), indicating their enormous untapped potential for these lands [3]. Utilizing *Miscanthus × giganteus* as ecosystem engineer on marginal lands minimizes competition with food crops while also improving poor soils through increased carbon sequestration and retention [1]. Research on marginal lands has emerged in the framework of phytoattenuation, which combines phytoremediation of the land with the production of a commercially viable energy biomass product for future development [[4], [5], [6], [7]]. Considering the vulnerability of *Miscanthus* sp. during the first two years of establishment [8], marginal lands may require additional support to meet the plants’ nutrient needs and ensure seedling survival in temperate climates. The need to develop fertilization strategy for *Miscanthus* sp. plantation in the first year was additionally highlighted by Yost et al. [9].

Organic additions [10] and biochar [11] can affect soil conditions and plant nutrition. Organic amendments such as sewage sludge, digestate, organic waste materials (including spent coffee grounds) contain readily available nutrients and can influence long-term biomass yields [12]. An estimated 8 million tons of spent coffee grounds (SCG) are produced globally each year [13], representing a significant potential resource for value-added applications. SCG may be valorized by thermal transformation, forming biochar for further application as a soil amendment [14]. Biochar has additionally been identified as a promising soil supplement for enhancing various soil characteristics and increasing agricultural productivity [15,16]. Biochar is formed by thermal transformation of organic matter in oxygen-limited conditions. Over the last decade, many feedstocks have been successfully used to produce biochar, and the positive role of biochar in soil has been documented, including increased soil fertility, water retention, improved microbial activity, and long-term carbon storage [[17], [18], [22]]. The persistence of biochar’s effects in the soil is a key issue; Joseph et al. [16] defined three types of biochar reaction times: short-term (1–3 weeks) – biochar dissolution, medium-term (1–6 months) – development of reactive surfaces, and long-term (>6 months) – biochar ageing. There is a need to investigate and document data on the diverse-term effects of biochar application so that guidelines for safe and effective soil application can be developed. Another important aspect of environmental effect is life-cycle assessment, which includes manufacture, transportation, and biochar field application [19].

This data article presents the findings of a greenhouse experiment with *Miscanthus × giganteus* planted on marginal soil with SCG and biochar additives. The data address a circular economy approach: phytoattenuation of marginal soil with *Miscanthus × giganteus* and the reuse of SCG and SCG-derived biochar. The medium-term effect of soil amendments was defined by incorporating 4 life-cycle stages: feedstock selection, biochar production, soil-application, and medium-term fate.

The objectives of this pot-scale experiment with energy biomass (*Miscanthus × giganteus*) cultivation on marginal soils with amendments were to: 1) collect data on *Miscanthus* growth response, photosynthesis parameters, and chlorophyll content index; 2) collect data on nutrients and caffeine concentrations in leachates; 3) collect data on the elemental composition of plant tissue and soil after the medium-term screening, as well as 4) present data on morphological changes in root tissues of *Miscanthus* after exposition.

## 3 Data Description

The data are stored in 5 datasets deposited in the Bridge of Knowledge Gdansk University of Technology repository:

Dataset 1: Nawrot, N., Tarasewicz, K., Strycharz, J., & Wojciechowska, E. (2024). The impact of raw and thermally processed spent coffee grounds on the *Miscanthus x giganteus* plantation (Versions 3.0, 1) [Dataset]. Gdańsk University of Technology. https://doi.org/10.34808/h744–9m76

**Fig. 1:**
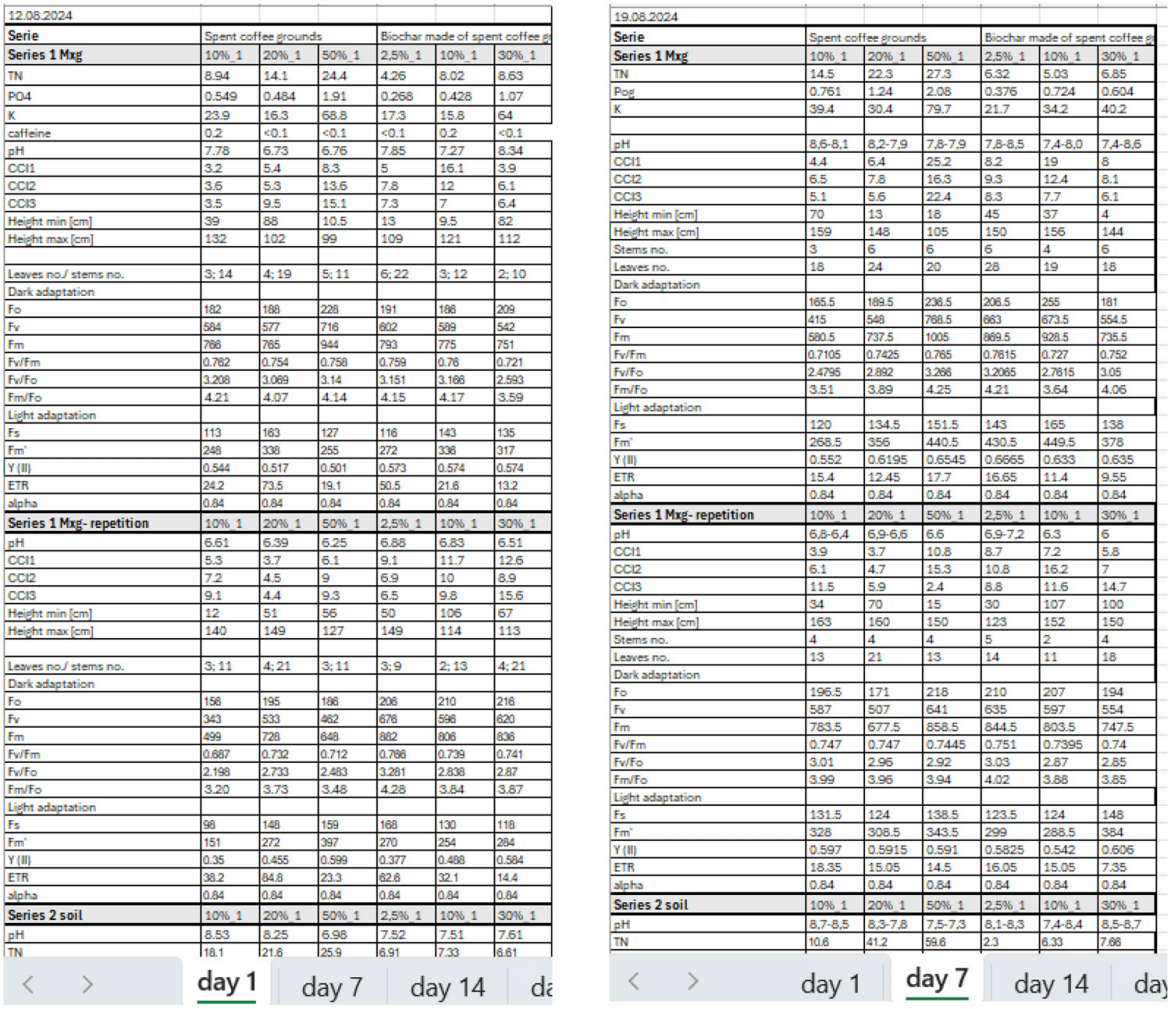
Structure of the table for data collection with nutrients concentration in leachates, growth parameters of *Miscanthus × giganteus*, and photosynthesis process parameters presented in Dataset 1.

This data collection includes all findings from a medium-term, pot-scale experiment in which *Miscanthus* was planted in marginal soil treated with SCG and biochar derived from SCG (see example in Fig. 1). Each day of the analysis (days 1, 7, 14, 21, 28, 35, and 42) is presented in a separate data table. The findings regarding caffeine and nutrient concentrations in pot leachates are shown. Results on plant growth and fluorimeter readings indicating the effectiveness of the *Miscanthus* photosynthesis process are also presented.

**Fig. 2:**
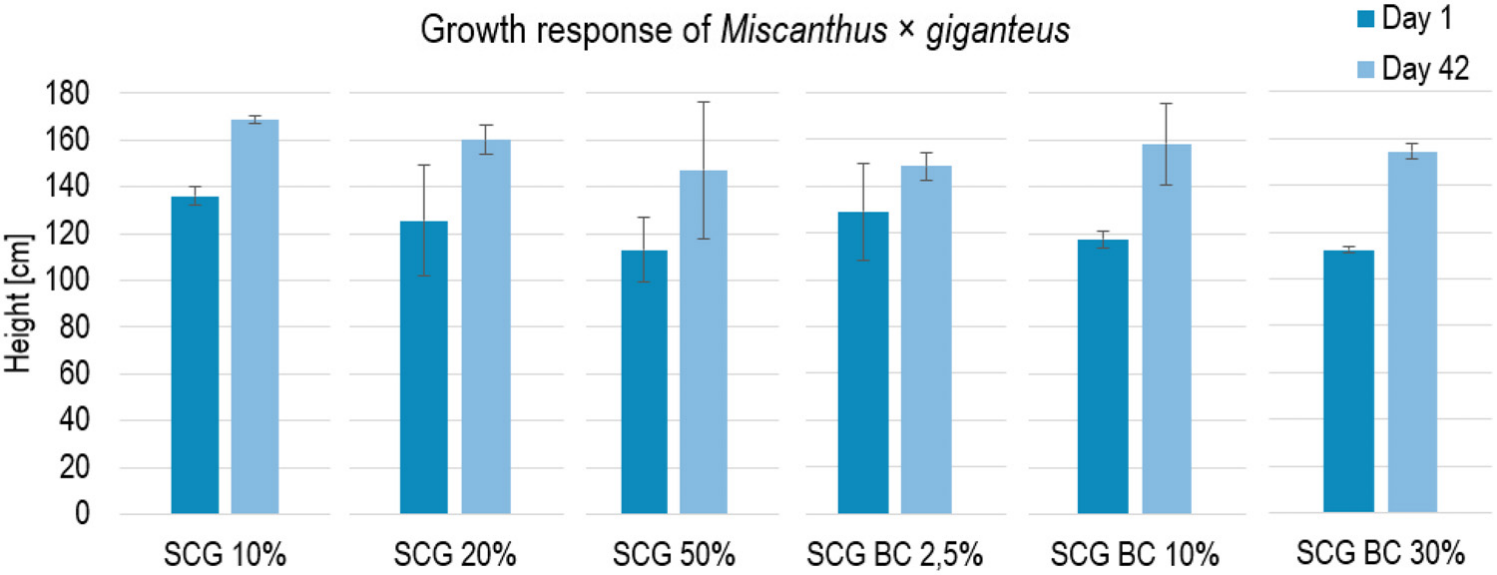
Growth parameters of *Miscanthus × giganteus* in diverse treatments; Explanations: SCG – spent coffee grounds, BC - biochar.

**Fig. 3:**
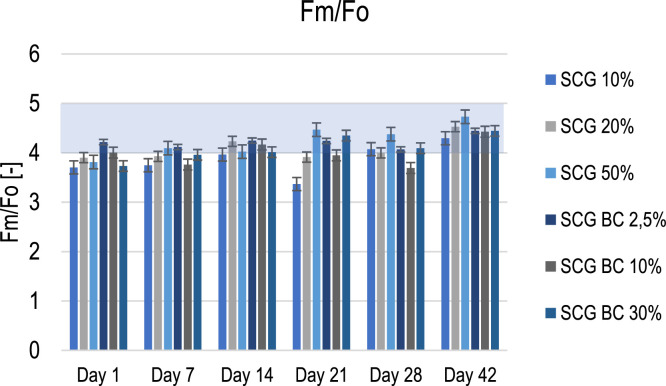
Maximum to initial fluorescence ratio; Explanations: blue area – optimum Fm/Fo ratio.

The data provide important information on *Miscanthus* responses to selected soil amendments, for example changes in plant growth over the experiment (Fig. 2) or photosynthetic parameters measured throughout the experiment (Fig. 3).

**Fig. 4:**
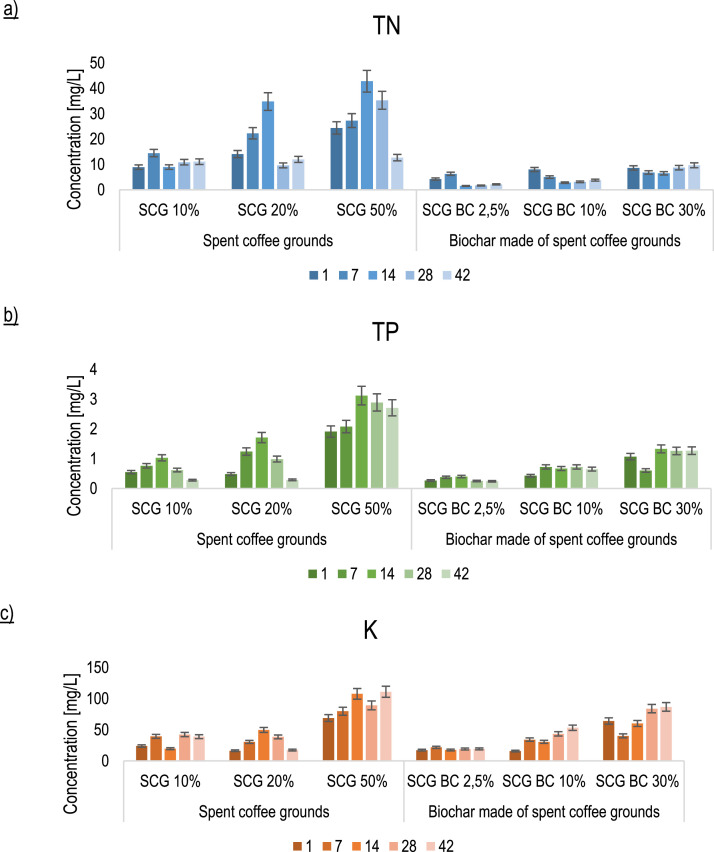
Nutrients concentrations [mg/L] in leachates from pots; Explanations: TN – total nitrogen, TP – total phosphorus, K - potassium.

Moreover, the changes in nutrient leaching from pots during the experiment were verified and provide information regarding the safe use of the selected amendments (Fig. 4). SCG supplementation showed a greater influence on the presence of TN, TP, and K in the leachates.

Dataset 2: Nawrot, N. (2025). The effect of raw and thermally processed spent coffee grounds on the development of *Miscanthus x giganteus* tissues (1–) [Dataset]. Gdansk University of Technology. https://doi.org/10.34808/h226-gv11

**Fig. 5:**
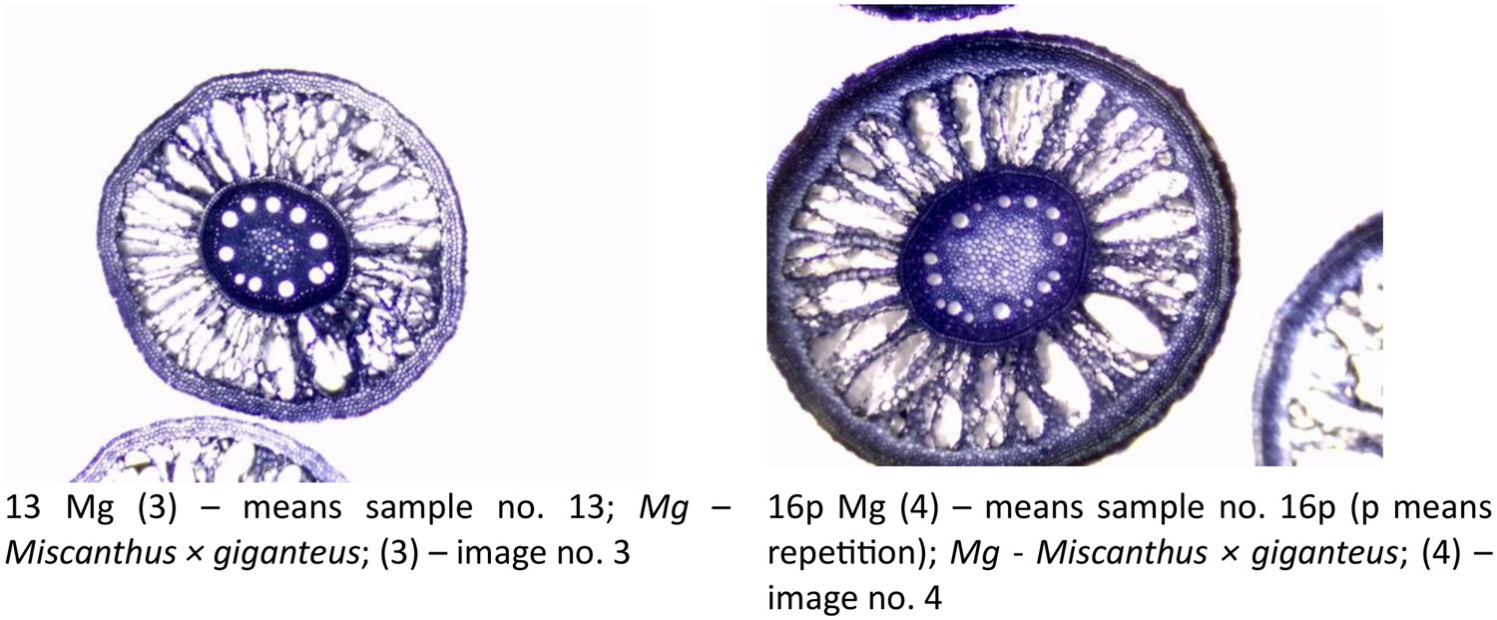
Example of images collected from microscopic observations and gathered in Dataset 2.

This dataset includes images of cross-sectioned *Miscanthus* roots collected at the end of the pot experiment with marginal soil mixed with SCG as well as SCG-derived biochar (see examples in Fig. 5). The dataset includes descriptions of the data as well as folders containing '.jpg' photos of *Miscanthus* root sections taken at the beginning and at the end of the experiment.

**Fig. 6:**
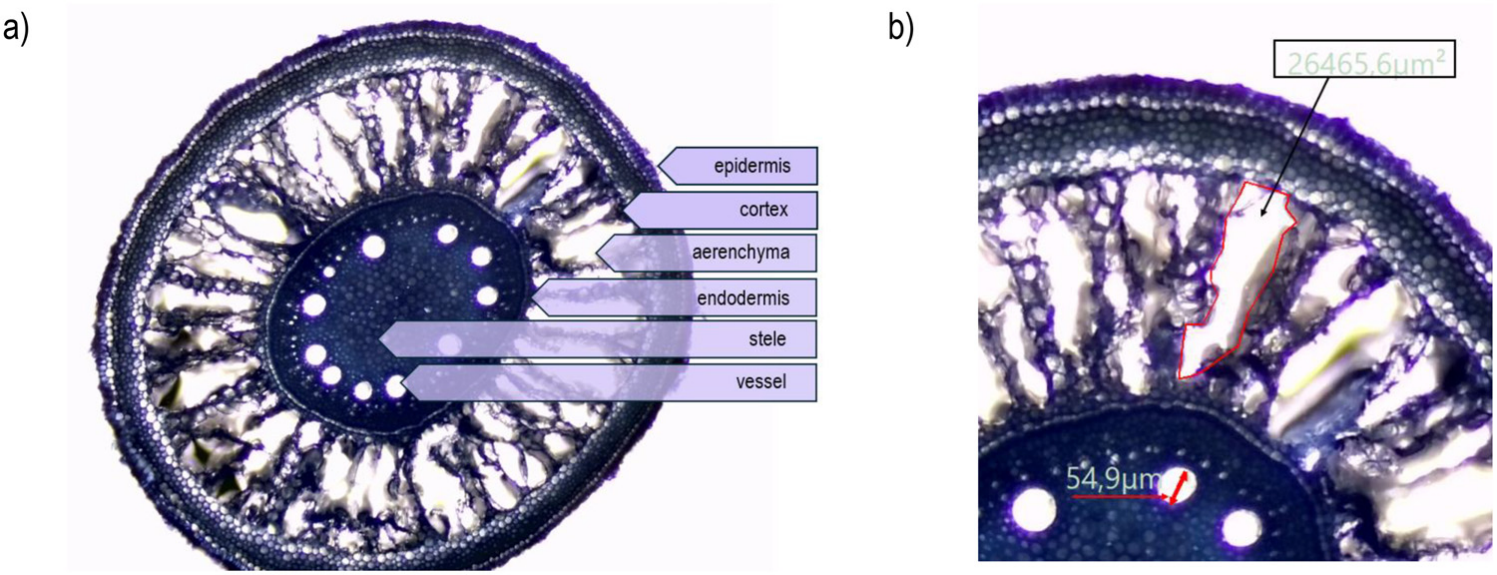
A method of describing and measuring microscopic data: a) tissue description and b) measurements performed with the DCTCamViewer software (Delta Optical, Poland; https://deltaoptical-dltcamviewer.software.informer.com/#google_vignette).

The data allows to determine the tissue development, including cortex, aerenchyma, epidermis, endodermis, stele, and vessels formation (Fig. 6a). The measurements of the tissue are possible with the DCTCamViewer software (Delta Optical, Poland; https://deltaoptical-dltcamviewer.software.informer.com/#google_vignette) (Fig. 6b).

Dataset 3: Nawrot, N., Strycharz, J., & Wojciechowska, E. (2025). Elemental composition of *Miscanthus giganteus* cultivated on soil amended with processed spent coffee grounds (1–) [Dataset]. Gdańsk University of Technology. https://doi.org/10.34808/0yfx-ep80

**Fig. 7:**
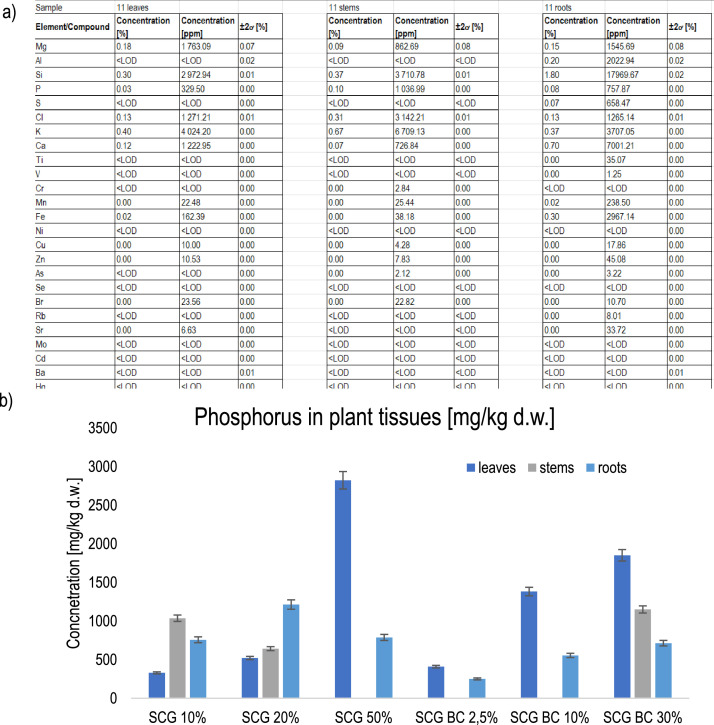
Example of: a) elemental composition of *Miscanthus × giganteus* in sample 11 (leaves, stems, and roots) presented in Dataset 3 and b) phosphorus concentrations [mg/kg d.w.] in parts of the plant (leaves, stems, and roots) in different treatments; the ±2 standard deviations (±2 SD) ranges from 2 % to 7 %.

This dataset contains the elemental composition results of *Miscanthus* roots, stems, and leaves (example presented in Fig. 7a). Samples were collected after medium-term screening (42 days) of *Miscanthus* planted in marginal soil with various soil amendment ratios (for SCG: 10 %, 20 %, and 50 % of SCG added into soil and for SCG-derived biochar: 2.5 %, 10 %, and 30 % v/v). Elements such as Mg, Al, Si, P, S, Cl, K, Ca, Cr, Mn, Fe, Ni, Cu, Zn, As, Mo, Cd, and Pb were analysed and may be compared across all tested elements and treatments (see example Fig. 7b for P).

Dataset 4: Nawrot, N., & Strycharz, J. (2025). Effects of Raw and Thermally Processed Spent Coffee Grounds on Miscanthus × giganteus Plantation: soil parameters (1–) [Dataset]. Gdańsk University of Technology. https://doi.org/10.34808/r6ax-5b22

**Fig. 8:**
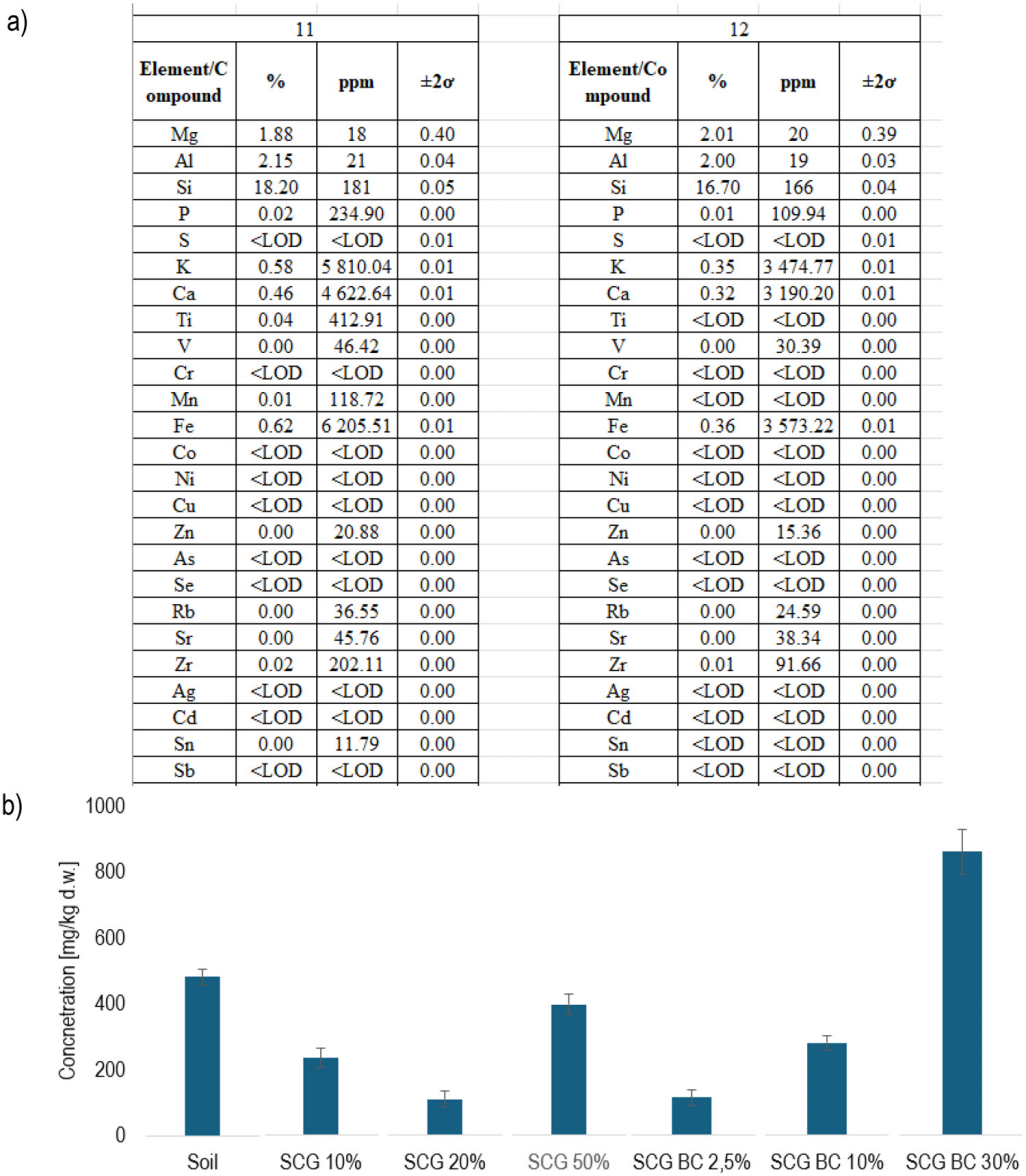
Example of: a) elemental composition of soil in samples 11 and 12 presented in Dataset 4 and b) phosphorus concentration [mg/kg d.w.] in soil at the beginning of the experiment and treatments at the end of the experiment.

This dataset contains the elemental composition results for soil at the end of the medium-term screening experiment (example presented in Fig. 8a). In addition, the initial parameters of marginal soil used in the experiment and SCG are presented. Elements such as Mg, Al, Si, P, S, Cl, K, Ca, Cr, Mn, Fe, Ni, Cu, Zn, As, Mo, Cd, and Pb were analysed and may be compared within all tested elements and treatments (see example Fig. 8b for P).

Dataset 5: Nawrot, N., & Strycharz, J. (2025). Growth response of *Miscanthus × giganteus* on spent coffee grounds and biochar supplementation into marginal soil (1–) [Dataset]. Gdańsk University of Technology. https://doi.org/10.34808/w3p6–0622

**Fig. 9:**
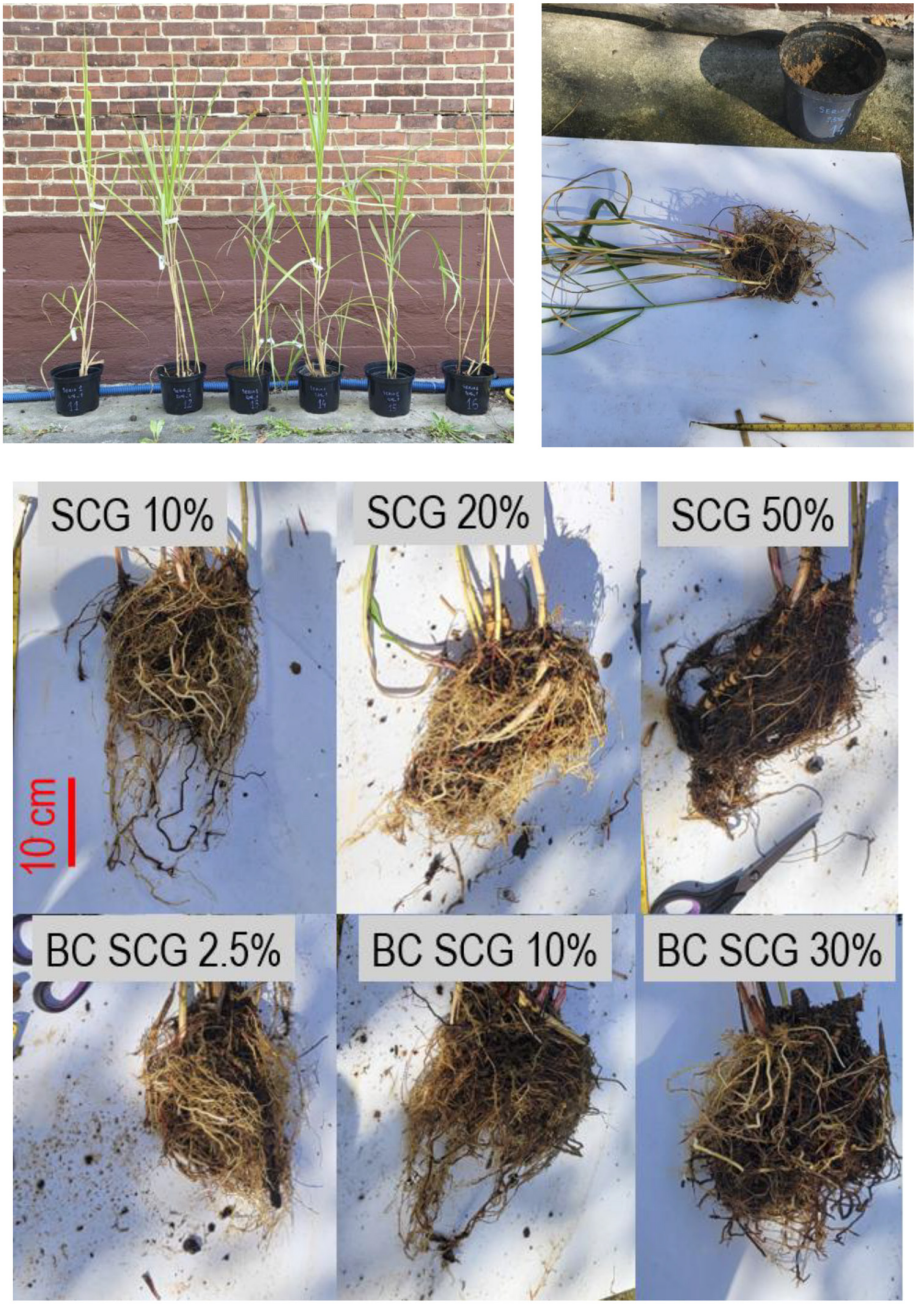
Images of *Miscanthus × giganteus* aboveground biomass developed during the experiment and roots at the end of the experiment (presented in Dataset 5).

This dataset is a collection of photos of the growth of the aboveground parts of the *Miscanthus* taken during the experiment. Images of measurements of the underground parts of the plants at the end of the experiment are also included (example presented in Fig. 9).

## 4 Experimental design, materials and methods

### 4.1 Experiment plan and duration

**Fig. 10:**
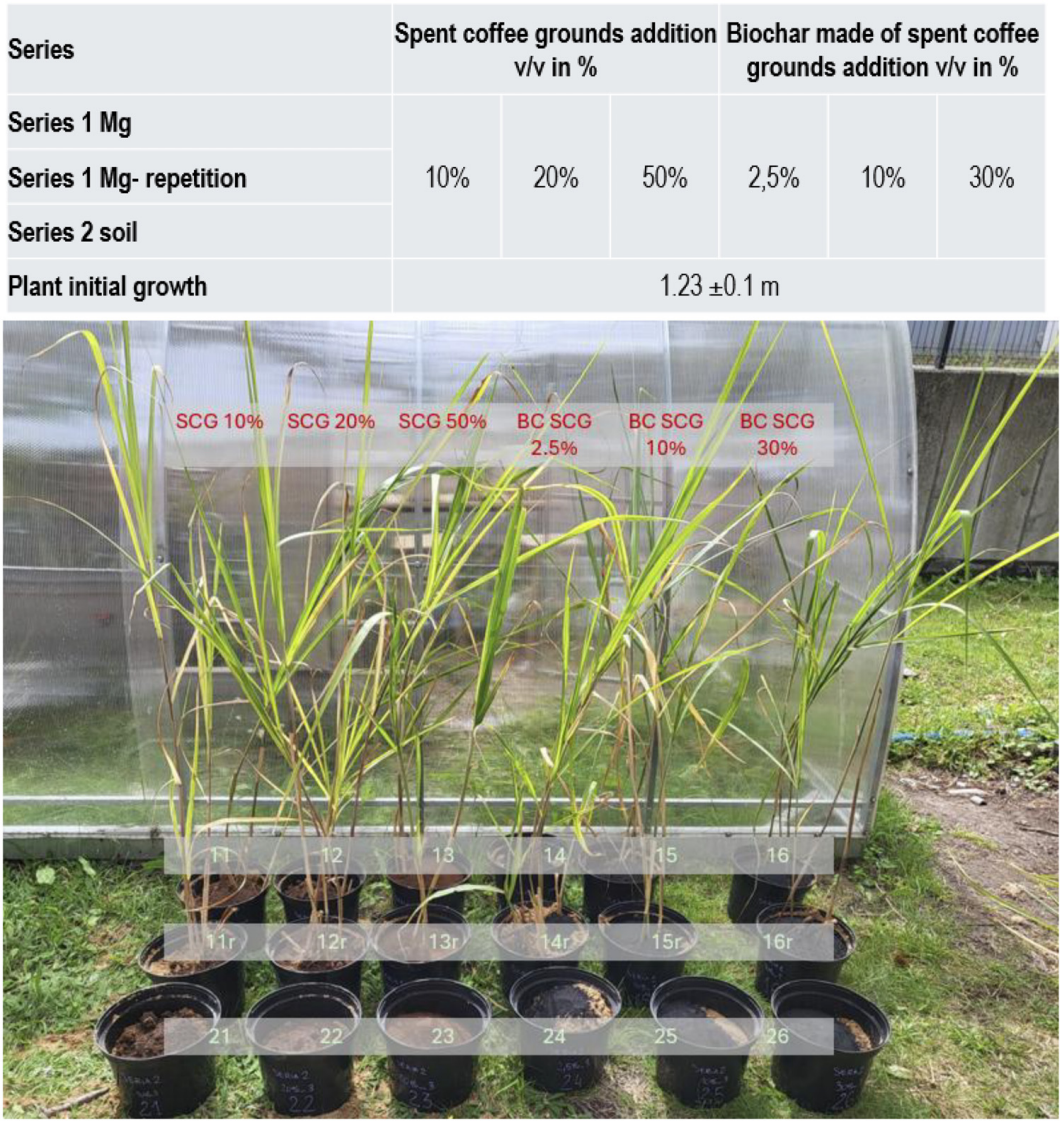
The view on the experimental pots at the beginning of the experiment; SCG – spent coffee ground; SCG – spent coffee grounds; BC - biochar.

The medium-term screening experiment was conducted in a greenhouse at Gdańsk University of Technology (54°22′08.8″N 18°36′48.5″E) under semi-artificial conditions on a pot scale (3 L pots). The experiment began on August 12, 2024 and ran until September 23, 2024. *Miscanthus × giganteus* (*M × g*) seedlings were delivered from the local plant nursery. At the beginning of the experiment, already cultivated, non-trimmed *M × g* seedlings were used, with an average height of 1.25 ± 0.1 m. The experiment was arranged in three series of pots as presented in Fig. 10. The first two series were designed as pots with marginal soil mixed with SCG or biochar made from SCG (marked hereafter as Series 1 *M × g* and Series 1 repetition) with *M × g* planting. The last Series (Series 2 soil) was designed as soil mixed with SCG or biochar made from SCG (without plants). In each series, SCG was added to the soil in proportions of 10 %, 20 %, and 50 % v/v (in total 3 pots per series). Biochar made from SCG was added in a proportion of 2.5 %, 10 %, and 30 % v/v (in total 3 pots in series). In total, 18 plots were monitored during the experiment. Irrigation was performed twice a week (about 0.7 L of tap water was used per pot; this volume was established based on soil moisture). The pots had holes at the bottom to allow water to percolate freely. The leachates were collected in plastic containers put underneath the pots during irrigation.

### 4.2 Marginal soil collection and parameters

The marginal soil was collected in the Pomeranian area of northern Poland (54°20′13.1″N, 18°20′48.4″E). The region is characterized by Pleistocene fluvioglacial deposits, with sand and gravel dominating the soil profile. Historically, glacial and post-glacial processes formed this region, resulting in largely coarse-textured soils with low natural nutrient content and minimal organic matter accumulation. Although the area is not significantly industrialized, it is impacted by a variety of land uses, including agriculture, local transportation networks, and distributed small-scale industry. Overall, the site is potentially subject to low-to-moderate anthropogenic pressures. The top layer of soil (0–30 cm) from a marginal field was collected with a stainless-steel shovel and delivered to the Gdansk University of Technology campus (54°22′08.8″N 18°36′48.5"E). Macronutrients and elemental composition of marginal soil is presented in Dataset 4. The pH of collected soil was neutral (pH=6.7). The granulometric analysis indicated a sand composition (0.063–2.0 mm > 97 %). Elemental analysis was performed with non-destructive XRF method (Titan S1, Bruker, USA) using the ‘Soil’ Calibration.

### 4.3 Soil amendments

Spent coffee grounds (SCG) were gathered from the waste bin of the coffee machine. Fresh material was immediately dried in a laboratory dryer at 40 °C and stored in a container for use in the experiment. The basic CHN properties of this feedstock were as follows: *C* = 47.7 %, *H* = 7.0 %, *N* = 0.35 %, and H/*C* = 0.15. The elemental composition is presented in Table 1.

**Table 1:** Elemental composition of spent coffee grounds utilized as soil amendment.

Element	Concentration [ppm]	Standard deciation ±2ơ [ppm]	Element	Concentration [ppm]	Standard deciation ±2ơ [ppm]
**P**	283	29.2	**Sr**	6.72	5.23
**S**	1871	127	**Zr**	<LOD	-
**Ca**	2830	57.4	**Ag**	<LOD	-
**Ti**	<LOD	-	**Cd**	<LOD	-
**V**	<LOD	-	**Sn**	<LOD	-
**Cr**	<LOD	-	**Sb**	<LOD	-
**Mn**	<LOD	-	**Ba**	<LOD	-
**Fe**	302	23.9	**Hg**	<LOD	-
**Co**	<LOD	-	**Tl**	<LOD	-
**Ni**	<LOD	-	**Pb**	<LOD	-
**Cu**	30.3	7.50	**Mg**	21,990	4205
**Zn**	32.9	7.91	**Al**	27,171	207
**As**	<LOD	-	**Si**	153,161	70.6
**Se**	<LOD	-	**K**	11,000	115
**Rb**	19.9	8.84			

A portion of the dried SCG was pyrolyzed at 500 °C in a batch reactor described by Kluska et al. [20] to produce biochar. The batch reactor consisted of a heating chamber with a crucible measuring 400 mm in length and 145 mm in diameter. The reactor's temperature was controlled by a thermocouple (T_control_) in the heating chamber. The measurement system used a PID controller to regulate the temperature in the heating chamber. The controller started the heater when the temperature dropped below 500 °C due to heat loss during the carbonization proces, and the heater was turned off when the temperature reached 500 °C. To reach a carbonization temperature of 500 °C, the heating chamber was initially set to 450 °C. After reaching 450 °C in the fixed bed, the chamber temperature was increased to 500 °C. Two thermocouples in the experimental crucible monitored the fixed bed's heating rate. The first thermocouple was 20 mm from the crucible wall, while the second was placed in the core. Biochar was characterized by *C* = 90.1 %, *H* = 3.7 %, *N* = 2.2 %, and H/*C* = 0.04.

### 4.4 Plant analyses

A centimetre tapeline was used to measure both the plant's aboveground and root sections. Growth parameters were gathered in Dataset 5 as a collection of photos taken regularly during the experiment. The photosynthesis parameters and chlorophyll content index (CCI) are presented in Dataset 1. Portable modulated fluorimeters (OSI-YII-MTR and OSI-FV-MTR) (Opti-Sciences, Hudson, USA) were used for *M × g* fluorescence measurements. The maximum quantum yield of photosystem II (Fv/Fm), the ratio of maximal fluorescence measured during the first saturation pulse to minimal fluorescence after dark adaptation (Fm/Fo), and the normalized measurement ratio that represents achieved efficiency of photosystem II under current steady-state photosynthetic lighting conditions (Y(II)) were determined according to standardized measuring protocols (Opti-Sciences, Hudson, USA). Portable chlorophyll meter (CCM-200 Plus, Opti-Sciences, USA) was used for chlorophyll content index (CCI) measurements.

The internal morphology of roots was investigated using randomly selected root samples from each pot. A 30 mm long segment of root was cut 50 mm from the root base and immersed in tap water. Roots were sectioned free-hand using a double-edge razor blade to create semi-thin (0.5–1 mm) slices. Cross section slices were placed in a Petri plate containing redistilled water (Milli-Q Ultrapure Water System, Merck). After soaking in water for 2–3 min, the cross-sections were submerged in toluidine blue dye for one minute [21]. After staining, the slices were washed in redistilled water, placed on a clean glass slide with a drop of water, and protected with a coverslip. Observations were conducted using an optical microscope (Delta Optical Genetic Pro with 3MP camera; Delta Optical, Poland) with lens magnifications of 4x and 10x, as well as an ocular magnification of 10x. Measurements were performed using DLT-Cam Viewer software (DeltaOptical, Poland).

Elemental analysis was performed with non-destructive XRF method (Titan S1, Bruker, USA) with the ‘Nutrients plant’ Calibration.

### 4.5 Leachates analyses

Leachates from plots were collected and immediately analyzed for total nitrogen, total phosporus, and potassium using the cuvette test (Hach Lange, USA). The standard procedures described by EN ISO 11,905–1, ISO 6878–1–1986, DIN 38,405 D11–4, and the Kalignost method were used. Thermostat HT 200S (Hach Lange, USA) and spectrophotometric device DR3900 (Hach Lange, USE) were utilized for sample mineralization and element measurement. Caffeine determination was performed in filterates (0.45 µm) using LC-MS.

## Limitations

The scale of the experiment is a limitation of this study; further investigations into the impact of soil amendments on *Miscanthus × giganteus* growth should be conducted in the field.

## Ethics Statement

Not applicable.

## Credit Author Statement

**Nicole Nawrot:** Methodology, Investigation, Writing – original draft; Writing – review & editing, Project curation; Resources; Supervision; **Jacek Kluska:** Methodology; Investigation.

## Data Availability

Bridge of KnowledgeElemental composition of Miscanthus giganteus cultivated on soil amended with processed spent coffee grounds (Original data)
Bridge of KnowledgeThe effect of raw and thermally processed spent coffee grounds on the development of Miscanthus x giganteus tissues (Original data)
Bridge of KnowledgeEffects of Raw and Thermally Processed Spent Coffee Grounds on Miscanthus × giganteus Plantation: soil parameters (Original data)
Bridge of KnowledgeGrowth response of Miscanthus giganteus on spent coffee grounds and biochar supplementation into marginal soil (Original data)
Bridge of KnowledgeThe impact of raw and thermally processed spent coffee grounds on the Miscanthus x giganteus plantation (Original data)
